# The relationships between emerging adults self-efficacy and motivation levels and physical activity: a cross-sectional study based on the self-determination theory

**DOI:** 10.3389/fpsyg.2024.1342611

**Published:** 2024-05-29

**Authors:** Yuexian Tao, Tao Xu, Xin Wang, Chengyi Liu, Yinyin Wu, Mingyue Liu, Ting Xiao, Xinze Qiu

**Affiliations:** ^1^School of Nursing, Hangzhou Normal University, Hangzhou, Zhejiang, China; ^2^Faculty of Health Sciences and Sports, Macao Polytechnic University, Macao, China; ^3^Department of Epidemiology and Health Statistics, School of Public Health, Hangzhou Normal University, Hangzhou, Zhejiang, China

**Keywords:** emerging adulthood, physical activity, structural equation modeling, self-efficacy, motivation levels, self-determination theory

## Abstract

**Objectives:**

The study aims to examine the associations between exercise self-efficacy, motivation, physical activity, and body composition among emerging adults.

**Design:**

Cross-sectional.

**Methods:**

A convenience sample of 147 emerging adults participated in the Releasing Weight (RELEW) project. The InBody720 analyzer was used to measure body composition, and the International Physical Activity Questionnaire-Short, the Shortened Physical Activity Self-Efficacy Scale, and the Treatment Self-Regulation Questionnaire were used to measure self-reported physical activity, self-efficacy, and motivation. Structural Equation Modeling was used to exam the complex relationships among multiple variables. in this study. The Partial least squares structural equation modeling analysis with bootstrapping in Smart PLS 3 was employed to explore the path coefficients and t-values for the relationships that were thought to exist. Significance was determined using a threshold of *p* < 0.05.

**Results:**

The mean age of 147 participants was 18.5 ± 1.87, of whom 51.7% were female, recruited for this study. Exercise self-efficacy has a significant positive correlation with exercise motivation (*r* = 0.220, *p* = 0.008) and physical activity (*r* = 0.279, *p* < 0.001). Exercise motivation does not demonstrate significant associations with physical activity (*r* = 0.094, *p* = 0.298). Utilizing SEM, the model explained 9.2% of exercise self-efficacy, 11.8% of physical activity, and 68.3% of body composition variance. Mediation analysis revealed that exercise self-efficacy partially mediated the relationship between exercise motivation and physical activity (β = 0.106, *t* = 2.538, *p* < 0.05), and physical activity partially mediated the relationship between exercise self-efficacy and body composition (β = −0.296, *t* = 4.280, *p* < 0.001).

**Conclusion:**

This study sheds light on the complex relationships among motivation, self-efficacy, physical activity and body composition during emerging adulthood. Our results highlight the mediating role of self-efficacy and its impact on physical activity behaviors, offering valuable insights for targeted interventions and policy development to improve health outcomes in this demographic.

## 1 Introduction

Emerging adulthood, occurring between adolescence and full adulthood, is a critical transitional phase for health behavior development, significantly impacting long-term wellbeing (Arnett, 2000). Engaging in physical activity during this period greatly affects long-term health outcomes and overall wellbeing. Research consistently shows that physical activity behaviors during emerging adulthood play a crucial role in shaping future health outcomes (van Sluijs et al., 2021). Studies demonstrate that individuals maintaining higher physical activity levels during this phase exhibit reduced risks of chronic diseases later in life (Posadzki et al., 2020; Hale et al., 2021). Moreover, physical activity during emerging adulthood correlates with improved mental health outcomes and enhanced cognitive abilities (Chekroud et al., 2018; Heissel et al., 2023). Despite these benefits, there’s a concerning trend of declining physical activity levels during the transition from adolescence to emerging adulthood (Corder et al., 2019). This decline is influenced by academic pressures, increased work commitments, changes in social environments, and shifts in priorities (Lee et al., 2012; Farooq et al., 2020). Personality traits and societal roles also contribute to increased sedentary behaviors during this period (Champion et al., 2023). Understanding and addressing this decline is crucial to mitigating immediate health risks and preventing long-term consequences.

Numerous studies emphasize the significant role of physical activity in influencing body composition during emerging adulthood (Barbour-Tuck et al., 2018; Collins et al., 2023). Higher physical activity levels are associated with more favorable body composition profiles, including lower adiposity and higher lean muscle mass, reducing the risks of chronic diseases later in life. The interplay between psychological factors like self-efficacy and motivation also influences physical activity behaviors (Cao et al., 2023; Medeiros et al., 2023). Even though these factors have been looked at separately in studies, there hasn’t been a full look at how they all affect physical activity behaviors, especially in young adults just starting to become adults. This makes it harder to get a full picture of how exercise engagement works during this changing time.

Structural equation modeling (SEM) and mediation analysis constitute the core methodologies utilized to investigate the relationships among variables in this study. SEM allows for the simultaneous examination of complex relationships between observed and latent variables, offering a robust analytical framework for testing theoretical models (Stein et al., 2017). This study utilizes latent variable modeling to represent unobservable constructs using multiple observed indicators. Specifically, self-efficacy, motivation, physical activity, and body composition are conceptualized as latent variables composed of various observed indicators. Therefore, by integrating SEM and mediation analysis into the study design, a comprehensive understanding of the mechanisms underlying the relationship between exercise motivation, exercise self-efficacy, physical activity, and body composition can be obtained within the specific demographic of emerging adults.

### 1.1 Theoretical framework

Self-determination theory (SDT), introduced by Deci and Ryan (1985), focuses on human motivation and personality development, highlighting intrinsic psychological needs like autonomy, competence, and relatedness. SDT categorizes motivation into intrinsic and extrinsic forms (Tóth-Király et al., 2022), with intrinsic motivation driven by internal factors like enjoyment, while extrinsic motivation involves external rewards or pressures. Individuals closely link intrinsic motivation to the need for competence, seeking effectiveness and mastery in their interactions (Ryan and Deci, 2020).

Self-efficacy, rooted in social cognitive theory (SCT), proposed by Bandura (1978), revolves around an individual’s belief in their capacity to effectively perform specific tasks or manage challenging situations. This belief in personal abilities and competencies directly correlates with the need for competence in SDT. An individual’s confidence and perceived capabilities strongly influence their sense of mastery and effectiveness in various activities, contributing to the fulfillment of the competence need as outlined by SDT (Bandura, 2012). Therefore, while self-efficacy may not fully encapsulate the entirety of competence needs, it is undeniably interconnected with and contributes significantly to fulfilling competence needs within the SDT framework.

The relationships between self-efficacy, intrinsic motivation, and the fulfillment of psychological needs, as elucidated by SDT and SCT, provide a comprehensive lens to explore the dynamics of physical activity engagement among emerging adults in our study. Therefore, the purpose of the original initial study was to aim to explore and elucidate the relationships between self-efficacy and motivation on physical activity behaviors among emerging adults, specifically whether exercise motivation mediates the link between exercise self-efficacy and physical activity. However, during the analysis process, this study initially tested the model with motivation as a mediator and found it was a poor fit to the data. The absence of a good fit under the traditional formwork of SDT highlights the nuanced pathways through which motivational factors impact actual behavioral outcomes.

While SDT has conventionally proposed a hierarchical model where the satisfaction of psychological needs drives motivation, contemporary empirical findings indicate a more intricate and reciprocal relationship between self-efficacy and motivation. Recent empirical research has provided further support for the notion of a reciprocal relationship between self-efficacy and motivation within the framework of SDT. For instance, studies by Zhang et al. (2022) and Ma et al. (2023) have demonstrated that individuals with higher levels of self-efficacy exhibit greater motivation to engage in physical activity. Conversely, Trautner and Schwinger (2020) found that heightened motivation can positively influence self-efficacy beliefs, reinforcing the bidirectional nature of this relationship. This evolving understanding challenges the traditional hierarchical view within SDT, highlighting the dynamic interplay between self-efficacy and motivation in shaping behavior. Further research by Gryte et al. (2024) and Lev-Arey et al. (2024) underscores the importance of considering both self-efficacy and motivation as integral components in interventions aimed at promoting sustained physical activity participation among various populations. As a result, we redefined self-efficacy as the mediator.

**FIGURE 1:**
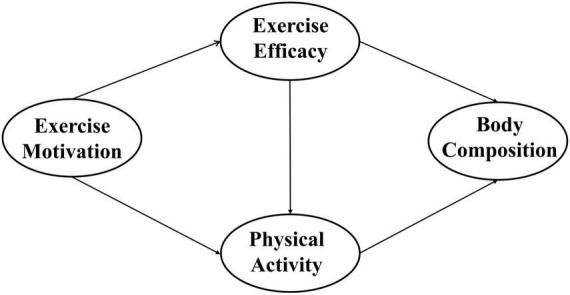
Motivation, self-efficacy and behavior-based self-determination theory model.

Therefore, this study aims to examine the interrelationship between self-efficacy and motivation and their collective influence on physical activity behaviors among emerging adults, with a particular focus on exploring how exercise self-efficacy mediates the link between exercise motivation and physical activity. This study’s hypotheses are as follows (see Figure 1):

• Exercise motivation is positively associated with exercise self-efficacy.
• Exercise motivation is positively associated with physical activity.
• Exercise self-efficacy is positively associated with physical activity.
• Exercise self-efficacy is positively associated with body composition.
• Exercise self-efficacy mediates the relationship between exercise motivation and physical activity.

## 2 Materials and methods

### 2.1 Study design

This study initially investigated the mediate of exercise motivation between exercise self-efficacy and physical activity in the framework of SDT and SCT. However, the SEM results indicated suboptimal fit indices for the proposed model, which explored the mediating role of motivation in the efficacy-physical activity relationship (the dataset is available in Supplementary File 1). Considering the intertwined relationship between motivation and efficacy, the positions of motivation and exercise efficacy were switched. The investigation was conducted at a university located in Hangzhou, China. This report follows the guidelines of Strengthening the Reporting of Observational Studies in Epidemiology (STROBE) (Ghaferi et al., 2021). The STROBE checklist can be found in Supplementary File 2.

### 2.2 Participants

#### 2.2.1 Sources of participant

The recruitment of participants was based on convenience sampling and was drawn from previous studies (Xu et al., 2023). The researchers distributed the research information sheet to students in common areas such as libraries, cafeterias, and student lounges in various departments and academic programs, ensuring representation from diverse backgrounds. Collaboration with faculty members and student organizations was also used to reach a wider pool of participants. The selection of participants was carried out by providing information about the study and its objectives to potential participants. Those who expressed interest and met the eligibility criteria were invited to participate in the study. The eligibility criteria for participants in this study included being (1) aged 18 to 25 years; (2) absence of any disease affecting participation in physical activity; and (3) willing to voluntarily participate in the study.

#### 2.2.2 Sample size

The sample size was determined based on the recommended guidelines for SEM (Wolf et al., 2013), which emphasize the need for an adequate sample size to ensure statistical power and reliable estimation of model parameters. The complexity of the SEM model, including the number of latent variables, observed variables, and paths to be estimated, was considered (Raykov and Marcoulides, 2012). The model is expected to have 14 estimated parameters. A general rule of thumb suggests that a minimum of 10–20 observations per estimated parameter is desirable to achieve stable and reliable estimates, resulting in a sample size of 140 (Bentler and Chou, 1987). Considering a possible 10% non-response rate and a potential invalid questionnaire, we used a convenience sampling method to recruit a total of 155 college students.

### 2.3 Data collection

The data collection phase took place concurrently with the recruitment phase, spanning from July 2023 to October 2023. During this period, a convenience sampling technique was employed to distribute 155 questionnaires among college students. Participants were provided with the questionnaires. The researchers ensured that the data collection process adhered to ethical guidelines and maintained participants’ confidentiality. The researchers emphasized the voluntary nature of participation and assured the confidentiality and anonymity of responses. The questionnaires were administered in a controlled environment (classrooms and designated research spaces) to minimize distractions and ensure accurate responses. Participants were given sufficient time to complete the questionnaires, and any queries or concerns were addressed promptly.

### 2.4 Measurement

The study collected data on various aspects, encompassing demographic characteristics, body composition, physical activity levels, self-efficacy, and motivation levels. To enhance the transparency of our methodology, the questionnaire utilized in this study has been made available as Supplementary File 3 or at the following link: https://osf.io/pbd53/.

#### 2.4.1 Demographic characteristics

Self-reported data was collected on demographic information: age, sex, school year (freshman, sophomore, junior, senior, etc.), location (urban vs. rural).

#### 2.4.2 Body composition

A height meter, securely fixed to a vertical surface, was used to measure the height. To ensure accuracy, the assistant repeated the height measurement twice. The average of the repeated measurements was used to minimize potential measurement errors. Body composition, which included body weight, body mass index (BMI), body fat (BF), percentage of body fat (BF%), waist-hip ratio (WHR), and visceral fat area (VFA), was collected using the analyzer InBody720 (Biospace, USA), which applies bioelectrical impedance analysis (BIA). The InBody720 offers a variety of advantages and accuracy in body composition testing (Park et al., 2016).

#### 2.4.3 Physical activity levels

Physical activity levels were obtained through self-reported measures provided by the participants. The self-reported data were collected using the International Physical Activity Questionnaire-Short (IPAQ-S) with 7 items, which had been translated into Chinese by Macfarlane et al. (2007). Participants were asked to report their frequency, duration, and intensity of exercise over the past week. The questionnaire collects information on the frequency, duration, and intensity of physical activity across various domains. The IPAQ-S provides a formula to calculate the total physical activity level. It involves multiplying the number of days per week by the total time spent per day for each activity category. The results are then summed to obtain the total time spent in physical activity per week. A sample item was “During the last 7 days, on how many days did you do vigorous physical activities like heavy lifting, digging, aerobics, or fast bicycling?” The scale demonstrates a retest reliability ranging from 0.66 to 0.88 (Macfarlane et al., 2007).

#### 2.4.4 Self-efficacy

The self-efficacy was obtained using the shortened physical activity self-efficacy scale (PASEESC) in simplified Chinese (Chen et al., 2019). The 8-item scale was assessed using a Likert scale ranging from 1 to 5, where 1 represents “disagree a lot” and 5 represents “agree a lot.” Participants with higher scores demonstrated a greater perception of self-efficacy. Furthermore, the effectiveness and consistency of the scale in evaluating psychological issues in Chinese teenagers have been proven, as indicated by a Cronbach’s alpha value of 0.87 (Zhou and Huo, 2022). The head of the scale reads, “Please evaluate to what extent you feel the following description fits you when participating in physical activity.” A sample item was “I can be physically active on most days of the week.” The reliability of the scales in this study was assessed using Cronbach’s α coefficient, which yielded a value of 0.92 (Chen et al., 2019).

#### 2.4.5 Motivation levels

The Treatment Self-Regulation Questionnaire (TSRQ) was used to determine participants’ motivation levels (Ryan and Connell, 1989). The TSRQ scale is a widely used tool to measure motivation for healthy behaviors (Teixeira et al., 2012). It includes a total of 15 items combined to form four component scores (i.e., individual autonomous motivation, internal regulation, external regulation, and no motivation), each of which has a range of (not at all true) 1 to 7 (very true) points. The sum of these four TSRQ component scores is the total TSRQ score, with higher scores indicating higher motivation levels. The head of the scale reads, “Regarding the extent to which you feel the following descriptions match you, please evaluate and judge the following 15 descriptions based on your actual feelings and experiences.” A sample item was “Because physical activity is very important for being as healthy as possible.” In this study, the Cronbach’s α of the four dimensions was 0.936, 0.827, 0.869, and 0.894, and the total reliability test of the scale is Cronbach’s α = 0.826 (Teixeira et al., 2012).

### 2.5 Ethical approval

The study has been approved by the ethical committee of Hangzhou Normal University (202259); all methods were performed in accordance with Helsinki guidelines. The voluntary nature of participation was emphasized throughout the entire process. The data collection process adhered to ethical guidelines. The confidentiality was assured by the anonymity of responses and the use of a case number.

### 2.6 Statistical analysis

Structural equation modeling served as the primary analytical technique to explore complex relationships among observed and latent variables. Latent variable modeling, a key aspect of SEM, allowed for the representation of unobservable constructs using multiple observed indicators, thereby capturing underlying dimensions inferred from observed variables. Self-efficacy, motivation, and physical activity were conceptualized as latent variables comprised of multiple observed indicators.

A mediation analysis was conducted to explore the indirect effects of motivation on physical activity through self-efficacy. An indirect effect refers to the influence that a predictor variable has on an outcome variable through an intervening variable. A direct effect, on the other hand, represents the influence of a predictor variable on an outcome variable without any intervening variables. The total effect encompasses both the direct and indirect effects, providing a comprehensive understanding of the overall impact of the predictor variable on the outcome variable (Baron and Kenny, 1986). The bootstrap method within the product of coefficients approach was employed to model the indirect effect, involving the multiplication of path coefficients representing relationships between the independent variable (motivation) and the mediator (self-efficacy) and between the mediator and the dependent variable (physical activity).

The partial least squares (PLS) method was chosen to construct the model due to its suitability for our sample size and skewed distribution, emphasizing predictive relevance over strict model fit to covariance structures (Cramer, 1993). Smart PLS (version 3) software facilitated the analysis of the structural equation model and the testing of theoretical hypotheses. Bootstrapping techniques, employing 5,000 resamples, were utilized within the product of coefficients approach to model the indirect effect. This involved multiplying path coefficients representing the relationships between the independent variable (motivation) and the mediator (self-efficacy), and between the mediator and the dependent variable (physical activity). Significance was determined using *t*-values exceeding 1.96 for a two-tailed test (Mackinnon et al., 2004).

Criteria for convergent validity included factor loading (> 0.708), Cronbach’s α (> 0.9), and composite reliability (CR, > 0.9) (Diamantopoulos et al., 2012; Hair et al., 2019). Convergent validity was evaluated by calculating the average variance extracted (AVE), which should exceed 0.5. Discriminant validity was assessed using the heterotrait-monotrait (HTMT) ratio of correlations, with thresholds below 0.85 indicating distinct constructs (Henseler et al., 2015). Structural models were evaluated based on the coefficient of determination (*R*^2^). *R*^2^ ranges from 0 to 1, with higher values indicating greater explanatory power to explain the data (Henseler et al., 2009) stated that *R*^2^ values of 0.75, 0.50, and 0.25 might be deemed significant, moderate, and weak.

It is important to note that the model should not have collinearity difficulties, as indicated by a variance inflation factor (VIF) of less than 5 (Becker et al., 2015). The predictive accuracy of the model was explained using the blindfolding-based cross-validated redundancy measure (*Q*^2^), which should have a value greater than 0 to indicate the predictive accuracy of this structural equation model. As a rule of thumb, *Q*^2^ values above 0, 0.25, and 0.50 indicate small, medium, and large predictive accuracies for PLS pathway models (Hair et al., 2019). The global fit of the PLS modeling was explained using the goodness-of-fit (GoF), with 0.1, 0.25, and 0.36 being the small, medium, and large values of the global fit of the model, respectively (Wetzels et al., 2009).

Additionally, several metrics associated with covariance-based SEM (CB-SEM) were computed to evaluate model fit to further enhance the evaluation of structural equation models, including the chi-square test statistic, Goodness of Fit Index (GFI), Comparative Fit Index (CFI), Tucker-Lewis Index (TLI), Incremental Fit Index (IFI), and Root Mean Square Error of Approximation (RMSEA), with established thresholds applied for assessment (Shi et al., 2019). Acceptable thresholds for these indices were as follows: GFI, CFI, TLI, and IFI values above 0.90 indicate good model fit (Bentler, 1990). RMSEA values below 0.08 indicate acceptable fit, with values below 0.05 indicating close fit (Browne and Cudeck, 1992).

The statistical analysis utilized SPSS (Statistical Product and Service Solutions, version 26). To describe the data, descriptive statistics were used. Either mean and standard deviation (mean ± SD) or median and interquartile range (IQR) were used, depending on how the Shapiro–Wilk test showed the data was distributed. Due to the skewed distributions of our variables, correlations were examined using the Spearman correlation coefficient (*r*). R (version 4.2.3) was utilized to visually represent correlations between variable dimensions, with syntax examples provided in Supplementary File 4.

## 3 Results

### 3.1 Demographics of the study population

The demographic characteristics of all participants are listed in Table 1. A total of 147 people were investigated, including 71 men and 76 women. Regarding the school year, the largest proportion of students were freshmen, making up 59.2% of the sample. In terms of home location, the majority of students (66%) reside in urban areas, while 34% are from rural areas. The weight categories of the participants revealed that 5.4% of students were classified as underweight, 15.7% fell within the healthy weight range, and a significant proportion of 78.9% were categorized as overweight or obese. 44.9% of students reported engaging in moderate-intensity activity, while 20.4% reported engaging in vigorous-intensity activity.

**TABLE 1:** Sociodemographic characteristics of the whole sample.

Variables	Frequency (%)/mean (standard deviation)/ (median [IQR])
**Age (mean ± SD)**	19.5 (1.87)
**Sex (%)**	
Male	71 (48.3)
Female	76 (51.7)
**School year (%)**	
Freshman	87 (59.2)
Sophomore	19 (12.9)
Junior	17 (11.6)
Senior	24 (16.3)
**Location (%)**	
Urban	97 (66.0)
Rural	50 (34.0)
**Weight categories (%)**	
Underweight	8 (5.4)
Healthy weight	23 (15.7)
Overweight/obesity	116 (78.9)
**Physical activity rating (%)**	
Inactive	51 (34.7)
Minimally active	66 (44.9)
HEPA active	30 (20.4)
**Physical activity levels (median [IQR])**	
Walking	247.5 [99, 594]
Moderate-intensity activity	160 [0, 360]
Vigorous-intensity activity	240 [0, 720]
Total (walking + moderate + vigorous)	1,010 [499, 1,657.5]
**Exercise self-efficacy (mean ± SD)**	23.48 (7.08)
**Exercise motivation (mean ± SD)**	67.82 (11.38)
**Body composition (mean ± SD)]**	
Height	164.88 (7.19)
Weight	58.19 (13.21)
Body mass index	21.27 (3.73)
Body fat	15.3 (7.29)
Body fat percentage	25.67 (8.05)
Waist-hip ratio	0.83 (0.07)
Visceral fat area	63.47 (36.48)
Metabolic equivalent	1,138.93 (801.99)

HEPA, health-enhancing physical activity; SD, standard deviation; IQR, interquartile range; physical activity level in MET-min/week, MET, metabolic equivalent; height in centimeters (cm); weight in kilograms (kg).

### 3.2 Bivariate correlations between all study variables

**FIGURE 2:**
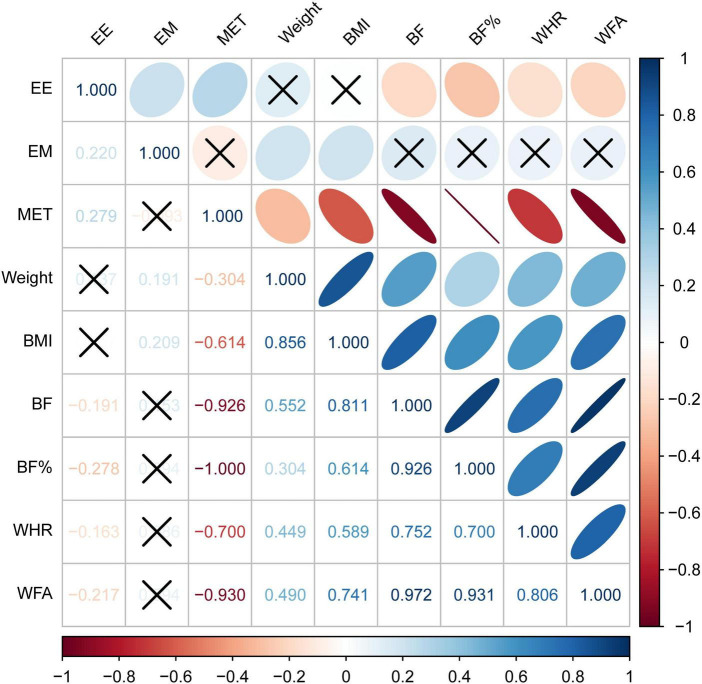
Correlations between exercise self-efficacy, exercise motivation, physical activity and body composition. Blue, positive correlation; red, negative correlation; dark color, high correlation; upper half, color plots; lower half, numerical plots; × , *p* > 0.05; EE, exercise self-efficacy; EM, exercise motivation; MET, metabolic equivalent; BMI, body mass index; BF, body fat; BF%, body fat percentage; WHR, waist-hip ratio; VFA, visceral fat area.

The Spearman correlation coefficient was utilized to assess the strength and direction of these relationships (Figure 2; Crawford, 2006) stated that *r* values of 0–0.19, 0.2–0.39, 0.40–0.59, 0.6–0.79, and 0.8–1 can be considered very weak, weak, moderate, strong, or very strong correlations. The statistical analysis revealed correlations among the variables under study. Exercise motivation has a positive correlation with exercise self-efficacy (*r* = 0.220, *p* < 0.05), indicating a relationship categorized as “weak.” However, no statistically significant relationships were observed between exercise motivation and physical activity (*r* = 0.094, *p* > 0.05), nor with various indicators of body composition such as weight, BMI, BF, BF%, WHR, and VFA. In contrast, exercise self-efficacy exhibited a positive correlation with physical activity (*r* = 0.279, *p* < 0.001), indicating a weakly strong relationship. Further examination focusing on physical activity uncovered a negative correlation with weight (*r* = −0.304, *p* < 0.001), body fat (*r* = −0.926, *p* < 0.001), and WHR (*r* = −0.700, *p* < 0.001), highlighting weak to very strong associations. Exercise self-efficacy displayed a negative correlation with several body composition indicators, including BF (*r* = −0.191, *p* < 0.05), BF% (*r* = −0.278, *p* < 0.05), WHR (*r* = −0.163, *p* < 0.05), and VFA (*r* = −0.217, *p* < 0.05). Table 2 shows the results of the Spearman correlation analysis between the main variables.

**TABLE 2:** Correlation coefficients of variables.

Variables	Exercise motivation	Exercise self-efficacy	Physical activity
Exercise motivation	1	–	–
Exercise self-efficacy	0.220**	1	–
Physical activity	0.279**	−0.093	1

**p < 0.01.

### 3.3 Testing the fit of the model

**FIGURE 3:**
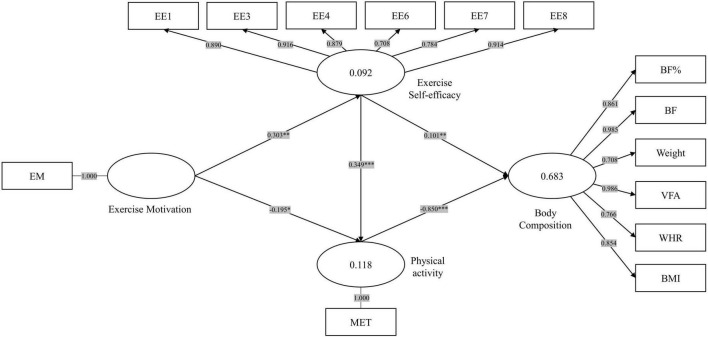
Structural equation modeling of exercise self-efficacy, exercise motivation, physical activity and body composition.

The reflective measurement model used factor loading, Cronbach’s, and CR coefficients to assess item reliability and internal consistency reliability. All factor loadings were greater than 0.708, as shown in Table 3, indicating that the model had acceptable item reliability. All Cronbach’s α and CR coefficients were above 0.90, indicating acceptable consistency and reliability (Diamantopoulos et al., 2012). All AVEs were greater than 0.5, and the results indicated that the model had acceptable convergent validity. The model depicted in Figure 3 was formulated using the PLS method implemented in Smart PLS 3.

**TABLE 3:** Factor loadings and convergent validity results.

Variables	Factor loadings	α	CR	AVE
**Exercise self-efficacy**		0.925	0.940	0.726
EE1	0.890			
EE3	0.916			
EE4	0.879			
EE6	0.708			
EE7	0.784			
EE8	0.914			
**Body composition**		0.935	0.947	0.750
BF%	0.861			
BF	0.985			
Weight	0.708			
VFA	0.986			
WHR	0.766			
BMI	0.854			

α, Cronbach’s alpha; CR, composite reliability; AVE, average variance extracted.

To further enhance the evaluation of our structural equation model, we computed several metrics associated with the CB-SEM. The chi-square (χ^2^) statistic was found to be 417.498 with 197 degrees of freedom, resulting in a ratio of χ^2^/df = 2.119. While the χ^2^/df ratio slightly exceeded the recommended threshold of 2, other fit indices showed acceptable values, such as the GFI at 0.797, the CFI at 0.909, the TLI at 0.894, and the IFI at 0.91. However, the RMSEA was found to be 0.088, slightly above the desirable cutoff of 0.08. Although the RMSEA 90% confidence interval ranged from 0.079 to 0.099, indicating some uncertainty around this estimate, the overall model fit was reasonable, with several indices indicating good fit, while the RMSEA slightly deviated from the ideal range.

The discriminant validity of the model was assessed using the HTMT ratios of the correlations, as shown in Table 4. The variables fall under different conceptual constructs. When the HTMT was < 0.85, it indicated that the discriminant validity of the model was acceptable (Henseler et al., 2015). Therefore, the measurement model used in this study demonstrated acceptable reliability and validity.

**TABLE 4:** Discriminant validity of the model.

Variables	1	2	3	4
1. Exercise motivation	–	–	–	–
2. Exercise self-efficacy	0.312	–	–	–
3. Physical activity	0.089	0.277	–	–
4. Body composition	0.164	0.177	0.765	–

Table 5 demonstrates the goodness of fit for the SEM. The coefficient of determination (*R*^2^) is a measure of the explanatory power within the model. The model’s independent variables explain a proportion of the dependent variable’s variance. The values indicated that the model was able to explain 9.2% of exercise self-efficacy, 11.8% of physical activity, and 68.3% of body composition, measured as weak and moderate levels of explanatory power (Henseler et al., 2009), respectively.

**TABLE 5:** Goodness of fit of the structural model.

Variables	SSO	SSE	SSE	*Q*^2^ (redundancy)	*Q*^2^ (communality)	*R* ^2^
		(overlapping)	(communality)			
Exercise motivation	147	147				
Exercise self-efficacy	882	827.720	407.552	0.062	0.538	0.092
Physical activity	147	131.714		0.104		0.118
Body composition	882	488.637	383.171	0.446	0.566	0.683

SSO, standardized solution output; SSE, standardized structural equation residuals; *Q*^2^ = 1-SSE/SSO; *Q*^2^(Redundancy) = 1-SSE(Overlapping)/SSO; *Q*^2^ = (Communality) = 1-SSE(Communality)/SSO.

Additionally, the blindfold-based cross-validation redundancy measure (*Q*^2^) provides an assessment of the precision of the structural model forecasts, indicating the extent to which the model can accurately predict the observed data. The analysis yielded *Q*^2^ values of 0.062 for exercise self-efficacy, 0.104 for physical activity, and 0.446 for body composition. These values suggest that exercise self-efficacy and physical activity have a weak impact on the structural model, while composition plays a significant role. The goodness-of-fit (GoF) assesses the quality of both structural and measurement models simultaneously. The estimated GoF value of 0.346 indicates a moderate fit of the model (Wetzels et al., 2009).

In Smart PLS 3, the conceptual model was analyzed using PLS-SEM with the bootstrapping approach. The results, as depicted in Table 6, reveal significant path coefficients and *t*-values for several paths. The regression coefficients are equivalent to direct effects (path coefficients adjusted for the presence of mediators). The pathways of exercise motivation–exercise self-efficacy and exercise motivation–exercise physical activity were statistically significant (β = 0.303, *t* = 3.243, *p* < 0.01; β = −0.195, *t* = 2.463, *p* < 0.05). The results showed that the pathways from exercise self-efficacy to physical activity were statistically significant (β = 0.349, *t* = 4.467, *p* < 0.001). Moreover, the pathways from physical activity (β = −0.850, *t* = 38.163, *p* < 0.001) and exercise self-efficacy (β = 0.101, *t* = 2.645, *p* < 0.01) to body composition were also significant.

**TABLE 6:** Analysis of regression relationship among variables.

Hypothesized paths	Standard path coefficients	*t*-value	95% CI
Exercise motivation → Exercise self-efficacy	0.303**	3.243	(0.105, 0.472)
Exercise motivation → Physical activity	−0.195*	2.463	(−0.343, −0.038)
Exercise self-efficacy → Physical activity	0.349***	4.467	(0.192, 0.498)
Physical activity → Body composition	−0.850***	38.163	(−0.902. −0.815)
Exercise self-efficacy → Body composition	0.101**	2.645	(0.031, 0.176)

Levels of statistical significance (***p < 0.001, **p < 0.01, *p < 0.05). The regression coefficients are the equivalent of direct effects (path coefficients adjusted for the presence of the mediators).

The results of the mediation effect test are displayed in Table 7. The direct effect of exercise motivation on physical activity is negative and statistically significant (β = −0.195, *t* = 2.463, *p* < 0.05). However, when considering the indirect effect (β = 0.106, *t* = 2.538, *p* < 0.05), the total effect (the sum of the direct and indirect effects) of exercise motivation on physical activity is negative but not significant (β = −0.089, *t* = 1.137, *p* > 0.05), suggesting that the presence of unmeasured mediators acting in the opposite direction to self-efficacy may reduce the overall beneficial effects of increased exercise motivation for increasing physical activity.

**TABLE 7:** Summary of mediation effect test results.

Effect	Path	Path coefficients	*t*-value	95% CI
Direct effect	Exercise motivation → Physical activity	−0.195*	2.463	(−0.343, −0.038)
Indirect effect	Exercise motivation → Self-efficacy → Physical activity	0.106*	2.538	(0.033, 0.200)
Total effect	Exercise motivation → Physical activity	−0.089	1.137	(−0.239, 0.065)
Direct effect	Self-efficacy → Body composition	0.101*	2.645	(0.031, 0.176)
Indirect effect	Self-efficacy → Physical Activity → Body composition	−0.296***	4.280	(−0.438, −0.162)
Total effect	Self-efficacy → Body composition	−0.196***	2.782	(−0.338, −0.055)

Levels of statistical significance (***p < 0.001, *p < 0.05).

Regarding the relationship between exercise self-efficacy and body composition, the total effect of exercise self-efficacy on body composition remains negative and significant (β = −0.196, *t* = 2.782, *p* < 0.01). Besides, the indirect effect through physical activity is negative and significant (β = −0.296, *t* = 4.280, *p* < 0.001), indicating that higher exercise self-efficacy is associated with better body composition. However, the direct effect is positive and statistically significant (β = 0.101, *t* = 2.645, *p* < 0.01), indicating that unmeasured mediators acting in the opposite direction to physical activity to reduce the overall beneficial effects of increased exercise self-efficacy for improving body composition.

## 4 Discussion

This study emphasized exercise self-efficacy as a significant factor influencing physical activity, particularly among young adults. Our findings extend this understanding by revealing negative correlations between exercise self-efficacy and various body composition measures, highlighting its potential impact on overall health outcomes. The observed positive relationship underscores the role of self-efficacy beliefs in influencing individuals’ decisions to initiate and sustain physical activity (Pan et al., 2022). Individuals with high self-efficacy levels, confident in their ability to perform physical tasks, are more likely to adopt and adhere to exercise routines, reflecting a proactive approach to maintaining an active lifestyle (Di Maio et al., 2021), which aligns with Bandura’s social cognitive theory and emphasizes the influential role of self-perceived capabilities in shaping behavioral outcomes. Moreover, the implications suggest that interventions targeting self-efficacy enhancement could effectively promote physical activity at both individual and community levels (Alshagrawi and Abidi, 2023). These findings contribute to the growing literature on the interplay between psychological factors and health behaviors, offering practical implications for interventions promoting sustained physical activity engagement. Additionally, our research has illuminated a noteworthy correlation between individuals’ motivation for engaging in physical activity and their self-efficacy beliefs. The observed correlation suggests that individuals with higher levels of motivation may concurrently exhibit enhanced self-efficacy, thereby fostering a positive feedback loop that potentially contributes to sustained engagement in physical exercise. Our results align with existing literature that posits a relationship between motivation and self-efficacy, emphasizing the dynamic nature of these constructs within the context of physical activity. Research has shown that exercise motivation not only positively predicts exercise climate and exercise self-efficacy but also plays an important role in predicting exercise behavior among college students (Zhao et al., 2023).

The absence of a direct correlation between motivation and physical activity was observed, with self-efficacy potentially mediating this association, which highlights the nuanced pathways through which motivational factors impact actual behavioral outcomes. Individuals with higher motivation levels demonstrate an increased likelihood of engaging in physical activity when coupled with elevated self-efficacy beliefs. Firstly, according to SDT, individuals are motivated to engage in behaviors that fulfill their basic psychological needs for autonomy, competence, and relatedness. In the context of exercise, individuals may possess high levels of intrinsic motivation, which arises from a genuine interest in and enjoyment of physical activity. However, their intrinsic motivation may not necessarily translate into actual exercise behavior if they lack confidence in their ability to perform the activities required (i.e., low exercise efficacy) (Rhodes et al., 2022). Thus, exercise efficacy acts as a mediator between intrinsic motivation and exercise behavior. Research supporting this notion has shown that individuals with higher levels of self-efficacy are more likely to autonomously engage in physical activity, as they feel competent in their ability to successfully complete exercise tasks (Teixeira et al., 2012; Brand and Cheval, 2019; Zhao et al., 2023). Besides, a study exploring the relationship between self-efficacy-mediated motivation and physical activity in patients with heart failure noted that after controlling for self-efficacy, the relationship between motivation and physical activity was no longer significant, suggesting full mediation (Klompstra et al., 2018). Secondly, socio-cultural factors such as societal norms and environmental constraints may impact individuals’ perceived self-efficacy in initiating and maintaining physical activity routines (Zhang et al., 2022). Moreover, individual differences in personality traits, such as trait self-esteem and neuroticism, may also moderate the relationship between motivation, self-efficacy, and physical activity behaviors (Kekalainen et al., 2020; Rhodes and Courneya, 2003). Finally, the temporal dynamics of motivation and self-efficacy, as well as the cyclical nature of physical activity engagement, may further complicate the direct association between these constructs (Rhodes and Dickau, 2012).

The finding of a positive and statistically significant path coefficient between exercise self-efficacy and body composition in the mediated effects analysis demands nuanced interpretation. It is essential to recognize the intricate interplay of factors underlying this relationship. Previous research has extensively documented the positive association between exercise self-efficacy and engagement in physical activity behaviors (Li et al., 2018; Zhang et al., 2024). Higher levels of self-efficacy are often linked to greater motivation and confidence in one’s ability to overcome barriers to exercise and adhere to exercise regimens (Schunk and DiBenedetto, 2021; Kekalainen et al., 2024). Consequently, individuals with elevated exercise self-efficacy may be more inclined to engage in physical activity, thereby potentially mitigating the adverse effects of poor body composition. However, the observed positive path coefficient between exercise self-efficacy and body composition’s direct effect may be confounded by the mediating role of physical activity. Physical activity serves as a crucial intermediary in the relationship between exercise self-efficacy and body composition. As individuals with higher exercise self-efficacy are more likely to engage in regular physical activity, the beneficial effects of exercise self-efficacy on body composition may be fully mediated through increased physical activity levels (Kim et al., 2017; Dishman et al., 2021).

While motivational factors are acknowledged as key contributors to health-related behaviors, the observed divergence invites further exploration into the interplay of psychological, social, and environmental influences that may modulate the impact of motivation on actual physical activity levels (Herrera et al., 2016). Thus, it is essential to consider the possibility of indirect pathways and moderating variables that shape the complex dynamics between motivation and behavioral outcomes. In addition, the interplay between motivation, self-efficacy, and subsequent physical activity levels underscores the importance of considering these intricate relationships in the design of interventions aimed at promoting and sustaining healthy behaviors. Recognizing self-efficacy as a mediator adds depth to our understanding of the motivational processes influencing physical activity, emphasizing the need for tailored strategies that enhance both motivation and self-efficacy to foster effective behavior change.

In summary, while this revised model may not entirely align with the traditional SDT framework, it represents an empirical understanding of the dynamics at play in our specific context of emerging adults’ physical activity behavior. Through this adaptation, we aimed to ensure that our model accurately reflected the observed relationships, thus contributing to a deeper comprehension of the factors influencing physical activity engagement among emerging adults.

## 5 Study limitations

Despite the study’s contributions, limitations exist that warrant consideration. Primarily, the cross-sectional nature of our research design poses constraints on inferring causality or establishing predictive relationships among the variables examined. While our study highlights associations between these constructs, caution must be exercised when assuming causal relationships. Additionally, the sample size and demographics might limit the generalizability of our findings. Considering the limitations posed by our sample size and demographics, we therefore applied PLS, which served as a valuable analytical approach for mitigating limitations associated with smaller sample sizes, non-normal distributions, or exploratory analyses. However, the constraints related to sample size and demographics, albeit mitigated to an extent by the chosen analytical method, still warrant careful consideration when interpreting and applying our results beyond the studied context. Moreover, the study acknowledges potential confounding biases that could impact the results. Sensitivity analyses were not conducted to investigate the potential effects of confounding biases, whether measured or unmeasured, on the results. Future research could consider employing sensitivity analyses to further explore these potential biases and their impact on the findings. Besides, the study recognizes the presence of social desirability biases and recall biases associated with self-report measures of physical activity. In our study, we attempted to mitigate these biases by employing validated self-reported measures and emphasizing the importance of providing honest and accurate responses. However, participants may have provided responses that they deemed socially desirable or may have inaccurately recalled their physical activity levels. The data collected at a single time point may not fully capture the dynamic and evolving nature of these factors over time. Future research could explore strategies to minimize social desirability and recall biases, such as employing ecological momentary assessment techniques or combining self-report measures with objective assessments. And longitudinal or experimental studies are warranted to have a better understanding of the temporal dynamics of self-efficacy, motivation, and physical activity among emerging adults.

Taken together, addressing the identified limitations could guide future research endeavors. Longitudinal studies are needed to examine the developmental trajectories of self-efficacy and motivation over time and their impact on sustained exercise engagement. Additionally, investigating potential moderating variables, such as social support or environmental factors, may provide further insights into the complex dynamics underlying motivational processes. Furthermore, applying advanced statistical techniques, such as longitudinal cross-lagged mediation modeling, can elucidate specific developmental and causal relationships among these constructs. By addressing these research gaps, future studies can advance our understanding of the motivational determinants driving physical activity behaviors and inform the development of more effective interventions targeting exercise promotion among emerging adults.

## 6 Conclusion

In conclusion, our study illuminates the dynamics between motivation, self-efficacy, and physical activity in emerging adulthood. These findings underscore the significance of self-efficacy as a mediator and exercise self-efficacy in influencing physical activity behaviors, providing a nuanced perspective for developing targeted interventions and policies to enhance health outcomes in this demographic.

## Data availability statement

The raw data supporting the conclusions of this article will be made available by the authors, without undue reservation.

## Ethics statement

Approval for this study was obtained from the Ethics Committee of Hangzhou Normal University (ratification date: November 14, 2022, 2022059). The studies were conducted in accordance with the local legislation and institutional requirements. The participants provided their written informed consent to participate in this study.

## Author contributions

YT: Conceptualization, Methodology, Project administration, Writing – original draft, Writing – review & editing. TaX: Conceptualization, Project administration, Visualization, Writing – original draft, Writing – review & editing. XW: Formal analysis, Methodology, Software, Writing – review & editing. CL: Writing – review & editing. YW: Formal analysis, Methodology, Writing – review & editing. ML: Investigation, Writing – review & editing. TiX: Investigation, Writing – review & editing. XQ: Investigation, Methodology, Writing – review & editing.
