# Supplementary material for: The relationships between emerging adults self-efficacy and motivation levels and physical activity: a cross-sectional study based on the self-determination theory
Source: Front Psychol. 2024 May 29;15:1342611. doi: 10.3389/fpsyg.2024.1342611 (PMC11168411; doi:10.3389/fpsyg.2024.1342611)
Supplement: Supplementary file 1 [file Data_Sheet_1.PDF]

Several metrics associated with covariance-based SEM (CB-SEM) were computed to evaluate model fit to further enhance the evaluation of structural equation model, including the chi-square test statistic, Goodness of Fit Index (GFI), Comparative Fit Index (CFI), Tucker-Lewis Index (TLI), Incremental Fit Index (IFI), and Root Mean Square Error of Approximation (RMSEA), with established thresholds applied for assessment (Shi et al.,2019). Acceptable thresholds for these indices were as follows: GFI, CFI, TLI, and IFI values above 0.90 indicate good model fit (Bentler,1990). RMSEA values below 0.1 indicate acceptable fit, with values below 0.05 indicating close fit (Browne et al.,1992).

This study investigates whether exercise motivation and exercise efficacy affect emerging adults' physical activity and the mediate of exercise motivation between exercise self-efficacy and physical activity. However, the SEM results reveal suboptimal fit indices for the proposed model investigating the mediating role of motivation in the efficacy-physical activity relationship. The chi-square statistic, while sensitive to sample size, yielded a significant value (Chisq=1400.105, df=249), indicating a potential mismatch between the model and observed data. Additionally, global fit indices, including the GFI, CFI, NFI, TLI, IFI, exhibited values below conventionally acceptable thresholds, with GFI=0.529, CFI=0.588, NFI=0.544, TLI=0.543, and IFI=0.592. The RMSEA further supported these findings, registering a high value of 0.177. These collective results suggest that the specified SEM model may inadequately capture the relationships under investigation. Moreover, the results of the indirect effect showed that this analysis did not yield statistically significant findings ($t = 1.793$, $p = 0.073$).

The model fit.

GFI	CFI	TLI	IFI	RMSEA
0.529	0.588	0.543	0.592	0.177

References:

Bentler, P. M. (1990). Comparative fit indexes in structural models. *Psychol. Bull.* 107(2), 238-246. doi: 10.1037/0033-2909.107.2.238

Browne, M. W., Cudeck, R. (1992). Alternative ways of assessing model fit. *Sociological methods & research*. 21(2), 230-258

Shi, D., Lee, T., Maydeu-Olivares, A. (2019). Understanding the model size effect on SEM fit indices. *Educ. Psychol. Meas.* 79(2), 310-334
