# Supplementary material for: The relationships between emerging adults self-efficacy and motivation levels and physical activity: a cross-sectional study based on the self-determination theory
Source: Front Psychol. 2024 May 29;15:1342611. doi: 10.3389/fpsyg.2024.1342611 (PMC11168411; doi:10.3389/fpsyg.2024.1342611)
Supplement: Supplementary file 3 [file Data_Sheet_3.PDF]

填写日期____________ 问卷编号：_____________

Fill in date____________ Questionnaire number：_____________

亲爱的受访者，感谢您抽出宝贵的时间参与我们的调查！您的意见对我们的项目至关重要。本次调查旨在了解初显期成人运动动机、运动效能和身体活动之间的关系，我们真诚期待您的真实回答。为了保护您的隐私，我们承诺**本次调查是匿名的**，并且您的**个人信息将被严格保密**。您的参与对于我们具有重要意义。如果您在填写问卷的过程中有任何疑问或需要进一步的信息，随时可以联系我们，您的反馈将受到高度重视。再次感谢您的参与，期待听到您宝贵的意见！诚挚。

Dear Respondents, thank you for taking your valuable time to participate in our survey! Your opinion is vital to our project. This survey aims to understand the relationship between exercise motivation, exercise efficacy and physical activity in emerging adults, and we sincerely look forward to your honest responses. In order to protect your privacy, we promise that this survey will be anonymous and **your personal information will be kept strictly confidential**. Your participation is important to us. If you have any questions or need further information while completing the questionnaire, feel free to contact us and your feedback will be highly valued. Thank you again for your participation and we look forward to hearing your valuable comments! Sincerely.

手　　机：__________

Mobile numbers:_______

性　　别：□男　　□女

S e x: □Male □Female

出生年月：__________

Year of birth:__________

年　　级：□大一　　□大二　　□大三　　□大四　　□其他________

School year: □freshman　□sophomore　□junior　□senior　□other

居 住 地：□城市　　□乡镇

Location: □urban　□rural

国际体力活动短问卷

International Physical Activity Questionnaire

引导语：我们希望了解人们在日常生活中进行的各种体育活动。这个问卷会问你在最近 7 天花在身体活动的时间，请回答每一个问题. 即使您并不认为自己是一个喜欢运动的人，请想一想你在工作时、做家务或者庭院劳动、从一个地方到一个地方以及在业余时间进行娱乐、锻炼或运动时所做的活动。

Head of the scale: We are interested in finding out about the kinds of physical activities that people do as part of their everyday lives. The questions will ask you about the time you spent being physically active in the last 7 days. Please answer each question even if you do not consider yourself to be an active person. Please think about the activities you do at work, as part of your house and yard work, to get from place to place, and in your spare time for recreation, exercise or sport.

想一想您在**过去 7 天内**所做的所有剧烈活动。**剧烈体力活动**是指需要耗费大量体力并使您的呼吸比正常情况下困难得多的活动。比平时更费力的活动。只想那些您每次至少进行了 10 分钟的体力活动。每次至少 10 分钟。

Think about all the vigorous activities that you did in the **last 7 days**. **Vigorous** physical activities refer to activities that take hard physical effort and make you breathe much harder than normal. Think only about those physical activities that you did for at least 10 minutes at a time.

1、 **最近 7 天内**，您有几天做了**剧烈的体育活动**，比如**提重物、挖掘、有氧运动或是快速骑车**？

During the last 7 days, on how many days did you do vigorous physical activities like heavy lifting, digging, aerobics, or fast bicycling?

每周_____天

_____ days per week

☐ 无相关体育活动 →跳到问题 3

☐ No vigorous physical activities →Skip to question 3

2、 在这其中一天您通常会花多少时间在剧烈的体育活动上？

How much time did you usually spend doing vigorous physical activities on one of those days?

每天_____小时_____分钟

_____ hours per day_____ minutes per day

☐ 不知道/不确定

☐ Don't know/Not sure

想想您在**过去 7 天内**所做的所有适度活动。**中等强度**的活动是指需要中等强度的体力劳动，并使您的呼吸比正常情况下困难一些的活动。只考虑那些每次至少持续 10 分钟的体力活动。

Think about all the moderate activities that you did in the **last 7 days**. **Moderate** activities refer to activities that take moderate physical effort and make you breathe somewhat harder than normal. Think only about those physical activities that you did for at least 10 minutes at a time.

2、 最近 7 天内，您有几天做了**适度的体育活动**，比如提轻的物品、以平常的速度骑车或打双人网球？**不包括走路。**

During the last 7 days, on how many days did you do moderate physical activities like carrying light loads, bicycling at a regular pace, or doubles tennis? **Do not include walking.**

每周_____天

_____ days per week

☐ 无适度体育活动 →跳到问题 5

☐ No moderate physical activities →Skip to question 5

4、 在这其中一天您通常会花多少时间在适度的体育活动上？

How much time did you usually spend doing moderate physical activities on one of those days?

每天_____小时_____分钟

_____ hours per day_____ minutes per day

☐ 不知道/不确定

☐ Don't know/Not sure

想一想您在**过去 7 天**中花在**步行**上的时间。这包括在工作和家里，从一个地方步行到另一个地方，以及任何其他纯粹为了娱乐、运动、锻炼或休闲而进行的步行。

Think about the time you spent **walking** in the **last 7 days**. This includes at work and at home, walking to travel from place to place, and any other walking that you have done solely for recreation, sport, exercise, or leisure.

5、 最近 7 天内，您有几天是步行，且一次步行至少 10 分钟？

During the last 7 days, on how many days did you walk for at least 10 minutes at a time?

每周_____天

_____ days per week

☐ 没有步行 →跳到问题 7

☐ No walking →Skip to question 7

6、 在这其中一天您通常花多少时间在**步行**上？

How much time did you usually spend **walking** on one of those days?

每天_____小时_____分钟

_____ hours per day_____ minutes per day

☐ 不知道/不确定

☐ Don’t know/Not sure

最后一个问题是关于您**最近 7 天内**平日**坐着**的时间。包括工作时、在家时、做功课时和闲暇时的时间。这可能包括坐在办公桌前、拜访朋友、阅读或坐着或躺着看电视的时间。

The last question is about the time you spent **sitting** on weekdays during the **last 7 days**. Include time spent at work, at home, while doing course work and during leisure time. This may include time spent sitting at a desk, visiting friends, reading, or sitting or lying down to watch television

7、 最近七天内，工作日您有多久时间是坐着的？

During the last 7 days, how much time did you spend sitting on a week day?

每天_____小时_____分钟

_____ hours per day_____ minutes per day

锻炼自我效能问卷

The shortened physical activity self-efficacy scale

	**请评估在参加体育活动时，您觉得以下描述在多大程度上符合您？**<br>**Please evaluate what extent do you feel the following description fits you when participating in physical activity?**	**非常不同意**<br>**Disagree a lot**	**不同意**<br>**Disagree**	**不确定**<br>**Not sure**	**同意**<br>**Agree**	**非常同意**<br>**Agree a lot**
1	在每周大部分天数中，我会锻炼身体。<br>I can be physically active on most days of the week.					
2	我可以要求父母或其他成年人和我一起锻炼身体。<br>I can ask my parent or other adult to do physically active things with me.					
3	即使我可以看电视或玩电子游戏，我仍然可以在每周大部分天数中的空余时间锻炼身体。<br>I can be physically active during my free time on most days even if I could watch TV or play (sedentary) video games instead.					
4	即使室外非常炎热或寒冷，我仍然可以在每周大部分天数中锻炼身体。<br>I can be physically active on most days even if it is very hot or cold outside.					
5	我可以要求我最好的朋友和我一起在每周大部分天数中一起锻炼身体。<br>I can ask my best friend to be physically active with me on most days.					
6	即使在家里我仍然可以锻炼身体。<br>I can be physically active even at home.					
7	我可以锻炼身体是因为我知道如何锻炼。<br>I can do active things because I know how to do them.					
8	不管我有多忙，我仍然可以在每周大部分天数中的空闲时间锻炼身体。<br>I can be physically active during my free time on most days no matter how busy my day is.					

治疗性自我调节问卷

Treatment Self-Regulation Questionnaire

	**关于以下描述你觉得符合你的程度请您根据自己的实际感受和体会，对下面 15 项描述进行评价和判断，并在最符合的项目中打对号**<br>**Regarding the extent to which you feel the following descriptions match you please evaluate and judge the following 15 descriptions based on your actual feelings and experiences, and tick the item that best matches them.**	完全不同意	不同意	比较不同意	不确定	比较同意	同意	完全同意
1	因为运动对保持健康非常重要<br>Because physical activity is very important for being as healthy as possible.							
2	我个人认为运动对我的健康是最好的<br>Because I personally believe physical activity is the best thing for my health.							
3	因为我觉得我想对自己的健康负责<br>Because I feel that I want to take responsibility for my own health.							
4	运动是我非常想做的一个重要选择<br>Because physical activity is an important choice I really want to make.							
5	我仔细思考过，相信运动对我生活的很多方面都很重要<br>Because I have carefully thought about it and believe physical activity is very important for many aspects of my life.							
6	运动和我的人生目标一致<br>Physical activity is consistent with my life goals.							
7	如果我不运动，我会感到愧疚<br>Because I would feel guilty or ashamed of myself if I don’t participant in physical activity.							
8	如果我不运动的话，我会觉得自己很糟糕<br>Because I would feel bad about myself if I don’t participant in physical activity.							
9	我之所以想运动是因为我受到了其他人给我的压力<br>Because I feel pressure from others to participant in physical activity permanently.							
10	因为如果我不运动，有人会对我失望<br>Because others would be upset with me if I don’t participant in physical activity.							
11	因为我想让其他人看到我可以做到<br>Because I want others to see I can do it.							

12	因为我想要别人认可我<br>Because I want others to approve of me.							
13	实际上我并不想运动<br>I really don’t think about participating in physical activity.							
14	我真的不知道为什么要这么做<br>I don’t really know why.							
15	因为按照别人说的去做比考虑运不运动要容易得多<br>Because it is easier to do what I am told than think about participating in physical activity or not.							
