# Supplementary material for: The relationships between emerging adults self-efficacy and motivation levels and physical activity: a cross-sectional study based on the self-determination theory
Source: Front Psychol. 2024 May 29;15:1342611. doi: 10.3389/fpsyg.2024.1342611 (PMC11168411; doi:10.3389/fpsyg.2024.1342611)
Supplement: Supplementary file 4 [file Data_Sheet_4.PDF]

The R code used for correlation analysis

```
install.packages("corrplot")
setwd("D:\\R\\GEE")
getwd()
library(readxl)
library(openxlsx)
library(geepack)
library(ggplot2)
library(corrplot)
library(ggcorrplot)
library(ggpubr)
mtcars <- openxlsx::read.xlsx("data.xlsx")
p_Jmtcars <- cor_pmat(mtcars, method='spearman')
cor_mtcars <- cor(mtcars, method = 'spearman')
corrplot(cor_mtcars, method = "ellipse", type = "upper", tl.col="black", p.mat=p_Jmtcars,sig.level
= 0.05, tl.cex=0.8, tl.srt = 45, tl.pos="lt")
corrplot(cor_mtcars, method = "number", type = "lower", tl.col=p, p.mat=p_Jmtcars, sig.level =
0.05, tl.cex=0.8, tl.pos="n", add=T,number.digits = 3,number.cex = 0.8,number.font = NULL )
```
